# Screening for Generalized Anxiety Disorder From Acoustic and Linguistic Features of Impromptu Speech: Prediction Model Evaluation Study

**DOI:** 10.2196/39998

**Published:** 2022-10-28

**Authors:** Bazen Gashaw Teferra, Sophie Borwein, Danielle D DeSouza, Jonathan Rose

**Affiliations:** 1 The Edward S Rogers Sr Department of Electrical and Computer Engineering University of Toronto Toronto, ON Canada; 2 School of Public Policy Simon Fraser University Vancouver, BC Canada; 3 Winterlight Labs Toronto, ON Canada; 4 Department of Neurology and Neurological Sciences Stanford University Palo Alto, CA United States; 5 The Centre for Addiction and Mental Health Toronto, ON Canada

**Keywords:** mental health, generalized anxiety disorder, impromptu speech, acoustic features, linguistic features, anxiety prediction, mobile phone

## Abstract

**Background:**

Frequent interaction with mental health professionals is required to screen, diagnose, and track mental health disorders. However, high costs and insufficient access can make frequent interactions difficult. The ability to assess a mental health disorder passively and at frequent intervals could be a useful complement to the conventional treatment. It may be possible to passively assess clinical symptoms with high frequency by characterizing speech alterations collected using personal smartphones or other wearable devices. The association between speech features and mental health disorders can be leveraged as an objective screening tool.

**Objective:**

This study aimed to evaluate the performance of a model that predicts the presence of generalized anxiety disorder (GAD) from acoustic and linguistic features of impromptu speech on a larger and more generalizable scale than prior studies did.

**Methods:**

A total of 2000 participants were recruited, and they participated in a single web-based session. They completed the Generalized Anxiety Disorder-7 item scale assessment and provided an impromptu speech sample in response to a modified version of the Trier Social Stress Test. We used the linguistic and acoustic features that were found to be associated with anxiety disorders in previous studies along with demographic information to predict whether participants fell above or below the screening threshold for GAD based on the Generalized Anxiety Disorder-7 item scale threshold of 10. Separate models for each sex were also evaluated. We reported the mean area under the receiver operating characteristic (AUROC) from a repeated 5-fold cross-validation to evaluate the performance of the models.

**Results:**

A logistic regression model using only acoustic and linguistic speech features achieved a significantly greater prediction accuracy than a random model did (mean AUROC 0.57, SD 0.03; *P<.*001). When separately assessing samples from female participants, we observed a mean AUROC of 0.55 (SD 0.05; *P*=.01). The model constructed from the samples from male participants achieved a mean AUROC of 0.57 (SD 0.07; *P=.*002). The mean AUROC increased to 0.62 (SD 0.03; *P<.*001) on the all-sample data set when demographic information (age, sex, and income) was included, indicating the importance of demographics when screening for anxiety disorders. The performance also increased for the female sample to a mean of 0.62 (SD 0.04; *P<.*001) when using demographic information (age and income). An increase in performance was not observed when demographic information was added to the model constructed from the male samples.

**Conclusions:**

A logistic regression model using acoustic and linguistic speech features, which have been suggested to be associated with anxiety disorders in prior studies, can achieve above-random accuracy for predicting GAD. Importantly, the addition of basic demographic variables further improves model performance, suggesting a role for speech and demographic information to be used as automated, objective screeners of GAD.

## Introduction

### Background

Anxiety disorders are characterized by an excessive and uncontrollable fear of what is to come and are associated with preparation for possible future adverse events [1]. Although anxiety is an important emotion that helps us prepare for future events, it limits the performance of day-to-day tasks when it becomes uncontrollable. Anxiety disorders are one of the most common mental health issues with an incidence of approximately 10% in the Canadian population [2]. Unfortunately, many Canadians affected by anxiety are unable to access psychological and psychiatric resources [3] due in part to the cost [4] and the general lack of availability [5]. Some of this deficit may be addressed using methods that automate certain aspects of the measurement and diagnosis of anxiety disorders.

In this study, we focused on generalized anxiety disorder (GAD) [6] and sought to automatically detect GAD from speech. We monitored speech because it is possible to passively and frequently sample ambient speech, ensuring that the privacy and confidentiality of the participants are appropriately handled. The capability to detect anxiety from ambient speech could be part of a system to automatically screen for anxiety, monitor treatment, and detect relapse.

We anticipated the following scenario for a system that included the capability to automatically predict anxiety from speech. Such a system would sample a sequence of the participant’s speech throughout the day and produce multiple predictions. Depending on the accuracy of individual predictions, the system could use multiple predictions to increase the overall accuracy of the final screening result. Note that this approach uses passively collected speech, which gives rise to its own challenges, including the need for a process of speaker identification to select words spoken by the participant.

Another motivation for pursuing the automatic detection of anxiety from speech is to avoid the subjectivity normally present in the screening and diagnosis of GAD. The current gold standard diagnosis for GAD is influenced by both the subject information supplied by a patient to a clinician and the subjective judgment of that information by the clinician. This subjectivity can lead to inaccurate diagnostic outcomes in patients [7]. There is a potential benefit of an objective marker of anxiety. In this study, we explored how well such a biomarker could be obtained from a person’s speech. Prior studies suggest that anxiety influences the acoustic features of speech [8], as these features are difficult for a person to control [9]. Moreover, anxiety may also manifest in the choice of words, which we refer to as linguistic features [10].

In an earlier study [11], we identified acoustic and linguistic features of speech that were correlated with anxiety as measured by the Generalized Anxiety Disorder 7-item (GAD-7) scale. Building on our earlier study and using the same participants and data, in this study, we aimed to build and measure the performance of a model that predicts whether participants are above or below the screening threshold for GAD on a much larger scale than what prior studies did. Previous studies validating the GAD-7 scale have shown that using a cut point of ≥10 optimizes sensitivity and specificity for identifying individuals with a diagnosis of GAD [12]. The model makes use of the previously identified features of speech together with the demographics of the participants.

This paper is organized as follows: the next subsection summarizes related work on anxiety prediction and proposes a hypothesis. The Methods section describes the speech sample collection methods, set of features used, and construction and evaluation of predictive models. The Results section presents the demographics of the participants and performance of the prediction model, while the Discussion section discusses the results and their implications for future research on anxiety detection.

### Related Work

Several previous studies have measured the association between speech features and various forms of anxiety, and other efforts sought to automatically detect anxiety from acoustic and linguistic features of speech. The studies explored in this section examine a broader class of anxiety disorders, including internalizing disorders, social phobia or social anxiety disorders (SADs), panic disorder, agoraphobia, and GAD.

McGinnis et al [13] identified several acoustic characteristics of speech that can be used to detect anxiety disorders in children. They studied the speech of 71 participants between the ages of 3 and 8 years and were able to detect internalizing disorders (a collective term for anxiety and depression). The authors extracted and selected several acoustic features from the speech produced in a 3-minute task based on the Trier Social Stress Test (TSST) for children [14]. These features included the zero-crossing rate, Mel frequency cepstral coefficients [15], zero-crossing rate of the *z* score of the power spectral density, dominant frequency, mean frequency, perceptual spectral centroid, spectral flatness, and the skew and kurtosis of the power spectral density. Several models were built to predict whether children had an internalizing disorder (43/71, 61%) or were healthy. Both logistic regression (LR) and a support vector machine (SVM) analyses [16] achieved a classification accuracy of 80%.

Weeks et al [17] found a relationship between anxiety and voice alterations. Their study showed a link between vocal pitch (characterized by fundamental frequency [F0]) and SAD. They collected impromptu speech samples from 46 undergraduate students, 25 (54%) with a diagnosis of SAD and 21 (46%) healthy controls. The participants also completed the Beck Anxiety Scale as a measure of self-reported anxiety severity [18]. Their results indicated that the mean F0 was positively correlated (*r*=0.72; *P*=.002) with anxiety severity in all male participants. However, the correlation in female participants was weaker (*r*=0.02; *P*=.92), indicating possible sex differences in the relationship between anxiety severity and vocal pitch. In a related continuing study [19], the authors attempted to classify men with SAD using the mean F0. Using a mean F0 value of 122.78 Hz, they achieved a sensitivity of 89% (8/9 male patients with SAD correctly classified) and a specificity of 100% (4/4 male healthy controls correctly classified).

Salekin et al [20] explored methods for detecting social anxiety and depression from an audio clip of a person’s speech. Their data set included a 3-minutes speech sample from each of the 105 participants describing what they liked and disliked about college or their hometown. The participants were asked to report their peak levels of anxiety during the speech. The authors presented and used a novel feature modeling technique called NN2Vec that can identify the relationship between a participant’s speech and affective states. Using the features from NN2Vec and a bidirectional Long Short-Term Memory Multiple Instance Learning network, they were able to detect speakers with high social anxiety with an *F*_1_-score of 90.1% and speakers with depression symptoms with an *F*_1_-score of 85.4%.

Baird et al [21] explored the effect of anxiety on speech by attempting to predict anxiety using sustained vowels. Their data set comprised 239 speakers (69 male participants) aged 18 to 68 years who performed various vocal exercises, which included sustained vowel sounds. They used the Beck Anxiety Inventory (BAI) [22] questionnaire as a label for each participant. The BAI is also one of the scales used to screen for GAD. They used 4 classes of sustained *(a)* vowels from each participant: a sad phonation, a smiling phonation, a comfortable phonation, and a powerful or loud phonation. From the sustained vowels, they extracted acoustic features such as the SD of F0, intensity, and harmonic-to-noise ratio. Using these features and a BAI label, they trained a support vector regressor with a linear kernel and used Spearman correlation between the predicted and the actual label to evaluate the performance of their model. They split their data into training and test sets and achieved a Spearman correlation of 0.243 on the test data set. They reported a better performance of a Spearman correlation of 0.59 when they only considered the group with high BAI scores, indicating that the symptoms of anxiety are more observable in individuals with high anxiety.

Rook et al [23] hypothesized that the worrying behavior in GAD comes from the verbal linguistic process. They attempted to predict GAD using only linguistic patterns. A total of 142 undergraduate students (56 male and 86 female participants) were recruited and asked to recall and write down an anxious experience during their university life. Each participant filled out the GAD-7 scale score and the behavioral inhibition/behavioral approach scale (BIS/BAS) [24]. The Linguistic Inquiry and Word Count (LIWC) [25] method was used to extract features from the texts written by the participants. Another set of features was also used by combining the LIWC features with BIS/BAS scores. Several machine learning models were explored, including SVM with linear kernel, LR, naïve Bayes, and random forest. Their results showed that all the models performed significantly better than a random model. In addition, better performance was obtained from all the models except the SVM when the LIWC and BIS/BAS features were used together as inputs compared with using only the LIWC features.

Di Matteo et al [26] examined the relationship between passively collected audio data and anxiety and depression. Their study continued for 2 weeks, where 84 participants installed an app on their smartphone that collected the average volume of sounds (the average of 15-second audio collected every 5 minutes) and the presence or absence of speech in the environment. They then extracted 4 environmental audio-based features: daily similarity, sleep disturbance (on all nights and weeknights only), and speech-presence ratio. Their results showed that none of the extracted features were significantly correlated with anxiety. However, these features were significantly correlated with depression: daily similarities (*r*=−0.37; *P*<.001), sleep disturbance on weeknights (*r*=0.23; *P*=.03), and speech presence (*r*=−0.37; *P*<.001).

Di Matteo et al [27] also explored the relationship between linguistic features of speech and anxiety. They used passively collected intermittent samples of audio data from participants’ smartphones, which they converted to text. The authors used the LIWC approach [25] to classify words into 67 categories. They calculated correlations using 4 self-report measures: SAD, GAD, depression, and functional impairment. They observed a significant correlation between words related to perceptual process (*See* in the LIWC) with SAD (*r*=0.31; *P*=.003) and words related to rewards with GAD (*r*=−0.29; *P*=.007).

In their third study, using the data collected from the 84 participants, Di Matteo et al [28] attempted to predict GAD, SAD, and depression from the smartphone-collected data. The features used in this study included daily similarity, speech presence, weeknight sleep disturbance, death-related words, number of locations visited, number of exits from home, screen use, and time in darkness. Although the models built on these features achieved an above-random prediction accuracy for SAD and depression, they did not observe above-random prediction accuracy for GAD.

Overall, prior studies suggest that it is possible to detect anxiety disorders from speech. However, the largest sample size among these previous studies was a total of 239, with an average of 115 participants, which limits the generalizability of the results. In addition, the number of participants might not be the only factor affecting generalizability. Apart from the studies by Di Matteo et al [28] and Baird et al [21], the prior studies were mostly limited to very specific demographics: McGinnis et al [13] focused on children; Weeks et al [17], Salekin et al [20], and Rook et al [23] focused on undergraduate students at a university or college.

We hypothesized that by recruiting a substantially larger cohort (N=2000) with broader demographic characteristics than that in prior studies, it is possible to achieve above-random prediction accuracy in screening for GAD using acoustic and linguistic features that have been previously suggested.

## Methods

### Data Collection

#### Recruitment and Demographics

We must note that the participants recruited and the data used in this study are the same as those in our earlier study [11], which focused solely on the correlations between acoustic and linguistic features of speech and the GAD-7. This study used those features and additional demographics to construct a predictive model. Participants were recruited from a nonclinical population using Prolific [29], a web-based human participant recruitment platform. The inclusion criteria for this study were an age range of 18 to 65 years, fluency in English, English as a first language, and at least 10 previous studies completed on Prolific, with 95% of these previous Prolific tasks completed satisfactorily (as labeled by the study author). The Prolific platform also provided several relevant demographics of the participants, including their age and income. The data set was also balanced for sex (50% female and 50% male).

Participants who completed the study were paid £2 (approximately CAD $3.41; US $2.74) for approximately 15 minutes of work. They completed the entire study remotely, using their PCs.

#### Study Procedure

Participants were recruited on Prolific for a 10- to 15-minute task implemented through a custom website. Our earlier paper on the correlates of anxiety [11] described the data collection procedure in detail. Parts of the data collection procedure that are relevant for the purpose of this study are described in the following sections.

On the Prolific platform, individuals who met the inclusion criteria were presented with the opportunity to participate in this study. Those who wished to participate clicked on the study link, which brought them to a consent form that described the procedure and goals of the study and provided information on data privacy. If a participant provided consent, a hyperlink brought them to an external web app that implemented the tasks described in further sections.

Participants were asked to fill out the standard GAD-7 questionnaire [12] described in more detail in the Anxiety Measures section. They were then asked to perform a speech task, which was recorded using their computer microphone. The speech task followed a modified version of the widely used TSST [30], which aimed to evoke a moderate amount of stress from each participant. Prior studies [31,32] have shown higher activation (cardiovascular, skin conductance, and plasma levels of norepinephrine and testosterone) in participants with relatively higher anxiety after exposure to moderate stress induced by the TSST.

In the modified version of TSST, participants were told to imagine that they were job applicants invited for an interview with a hiring manager. They were told to imagine that it was a job that they really wanted—their so-called “dream” job. They were given a few minutes to prepare—to choose their “dream” job—and to think about how they would convince an interviewer that they were the right person for that position. Participants were also told that the recorded video would be viewed by researchers studying their behavior and language. Participants were then asked to speak for 5 minutes, making the case for themselves to be hired for that dream job.

Note that in the original TSST [30], participants would normally deliver their speech in front of a live panel of judges. If a participant finished their delivery in <5 minutes, the judges in the original TSST design would encourage the participant to speak for the full 5 minutes. For example, a statement of encouragement in the original TSST was, “What are your personal strengths?” In the modified TSST, we implemented a similar method to encourage participants to speak for the full 5 minutes; when our system detected silence (the absence of speech for >6 seconds), it would display several different prompts inviting participants to keep speaking on different topics related to the task. Finally, the modified TSST only included the first part of the original TSST, not the second task, which involved the performance of mental arithmetic.

#### Anxiety Measures

We aimed to predict, based on features of the speech, if a participant is above or below the screening threshold for GAD based on the GAD-7 scale. The GAD-7 [12] scale is a 7-item questionnaire that asks participants how often they were bothered by anxiety-related problems during the previous 2 weeks. Although the 2-week time period suggests that the GAD-7 measures a temporary condition, this is in contrast to the fact that a GAD diagnosis requires a 6-month duration of symptoms [33,34]. However, the GAD-7 has been validated as a diagnostic tool for GAD using a cutoff threshold of 10, with a sensitivity of 89% and specificity of 82% [12]. Thus, we chose to use the GAD-7 threshold of 10 to obtain a binary label of GAD as the indicator of anxiety.

Each of the 7 questions on the GAD-7 has 4 options for the participant to select, indicating how often they have been bothered by the 7 problems in the scale. These options and their numerical ratings are as follows: 0=not at all, 1=several days, 2=more than half the days, and 3=nearly every day. The final GAD-7 score is a summation of the values for each question, giving a severity measure for GAD ranging from 0 (no anxiety symptoms) to 21 (severe anxiety symptoms).

### Separation of Data for Analysis

Certain demographic attributes were directly indicative of anxiety. For example, sex is known to influence the prevalence of anxiety [35]. In addition, both age [36] and income [37] influence anxiety. Owing to the strong effect of sex and our interest in analyzing the effect of anxiety on both the sexes separately, we created a separate data set for female and male participants, in addition to the combined data set.

### Inputs to the Classification Model

The inputs to our models were acoustic and linguistic features that were determined in a previous study [11] to have a statistically significant correlation with the GAD-7. These features were found to be correlated with the GAD-7 after controlling for demographic variables such as age, sex, and personal income. These features are presented in Table 1 as all-sample, female sample, and male sample data sets. The definitions of these features are presented in Multimedia Appendix 1.

In addition to the acoustic and linguistic features, we explored the use of demographic information, such as age, sex, and personal income, as input features to the model. We decided to use these demographics as features in the model because they were available to us from the Prolific recruitment platform [29]. Should the model be used as a diagnostic screener in the future, it should be possible to obtain these demographics.

**Table 1:** Correlation of statistically significant acoustic and linguistic features with Generalized Anxiety Disorder-7 item (GAD-7) scale—results taken from earlier study (N=1744).

Feature	Data set						
	All-sample		Female sample (n=862)			Male sample (n=882)	
	*r*	*P* value	*r*	*P* value	*r*		*P* value
AllPunc	0.13	<.001	0.14	<.001	0.13		<.001
WC	–0.12	<.001	–0.13	<.001	–0.12		<.001
Speaking duration	–0.12	<.001	–0.11	<.001	–0.13		<.001
Period	0.12	<.001	0.16	<.001	0.08		.02
assent	0.10	<.001	0.10	.004	0.11		.001
negemo	0.10	<.001	0.11	<.001	0.08		.01
relativ	–0.09	<.001	–0.09	.006	–0.10		.002
motion	–0.08	<.001	–0.10	.003	–0.07		.048
Shimmer	0.08	<.001	0.10	.004	0.07		.04
swear	0.08	<.001	—^a^	—	0.10		.004
anger	0.08	<.001	0.11	.002	—		—
mfcc_std_2	–0.08	.002	—	—	–0.09		.005
mfcc_std_3	–0.07	.002	–0.10	.002	—		—
focusfuture	–0.07	.003	—	—	–0.08		.02
mfcc_mean_2	–0.07	.004	–0.09	.01	—		—
adverb	–0.07	.004	–0.11	<.001	—		—
time	–0.07	.004	—	—	–0.10		.004
function	–0.07	.005	—	—	—		—
negate	0.07	.006	—	—	0.08		.01
prep	–0.06	.007	—	—	–0.08		.02
WPS	–0.06	.007	–0.07	.03	—		—
anx	0.06	.008	—	—	0.08		.01
f0_std	0.06	.01	—	—	0.07		.04
hear	0.06	.01	—	—	0.10		.003
mfcc_std_5	–0.06	.01	—	—	–0.09		.01
death	0.06	.01	0.07	.04	—		—
ipron	–0.06	.01	—	—	–0.07		.04
see	–0.06	.01	-0.09	.006	—		—
affect	0.06	.02	—	—	0.07		.04
i	0.05	.02	—	—	—		—
family	0.05	.02	—	—	0.08		.02
mfcc_std_4	–0.05	.03	—	—	–0.07		.04
sad	0.05	.03	0.08	.01	—		—
ppron	0.05	.03	—	—	0.09		.01
space	–0.05	.04	—	—	—		—
article	–0.05	.04	—	—	–0.08		.01
leisure	0.05	.04	—	—	0.10		.002
friend	0.05	.047	—	—	—		—
lpcc_std_6	—	—	–0.09	.008	—		—
lpcc_std_4	—	—	–0.09	.008	—		—
intensity_mean	—	—	–0.09	.01	—		—
mfcc_mean_1	—	—	–0.09	.01	—		—
Dic	—	—	–0.08	.02	—		—
power	—	—	0.07	.03	–0.09		.01
lpcc_std_10	—	—	–0.07	.03	—		—
intensity_std	—	—	–0.07	.03	—		—
lpcc_std_12	—	—	–0.07	.04	—		—
mfcc_mean_8	—	—	0.07	.04	—		—
percept	—	—	–0.07	.046	—		—
lpcc_mean_4	—	—	0.07	.049	—		—
Apostro	—	—	—	—	0.09		.005
Sixltr	—	—	—	—	–0.09		.01
mfcc_mean_5	—	—	—	—	–0.08		.02
mfcc_std_11	—	—	—	—	–0.07		.045
f1_mean	—	—	—	—	0.07		.047

^a^Not available because the correlation was not significant.

### Construction and Evaluation of Classification Models

In this study, we aimed to evaluate the performance of a binary classifier that predicts if a person’s speech sample is in the “anxious” or “nonanxious” class based on the features of speech. The binary classification label is determined by processing the GAD-7 scale (which ranges from 0 to 21 in value) into 2 classes, anxious (GAD-7≥10) and nonanxious (GAD-7<10), where 10 is a well-established screening threshold [12] for GAD.

An LR model was trained on the training data to make predictions between the 2 classes. The construction and evaluation steps were as follows. First, the input features were normalized, so that each feature would have a mean of 0 and an SD of 1. Next, the data were undersampled to equalize the representations from both the anxious and nonanxious classes. This avoided the problem of class imbalance, which, if occurred, caused low predictive accuracy for the minority class (which was the anxious class in our case). Therefore, samples were randomly selected and removed from the majority class until the majority class had an equal number of samples to the minority class.

The model construction and training step used 3 data sets: a training data set, which was used to train the model; a validation data set, which was used to select the best hyperparameters during training; and a test data set, which was used to evaluate the performance of the trained model using area under the receiver operating characteristic (AUROC) metrics. These data sets were created within each sampling of the cross-validation (CV) scheme described next.

The CV scheme used a nested resampling with 2-level nested CVs—one CV nested within another [38]. In the outer loop, the data were split into 20% test data and 80% training and validation data. In the inner loop, 80% of the training and validation data were further split into 20% validation data and 80% training data. The inner loop was repeated 5 times, each with a different sampling to obtain a different 20/80 split. For each such split, the best hyperparameters were selected to maximize the accuracy of the validation data after training on the training data of the inner loop. After selecting the best hyperparameters from the inner CV loop, training was once again performed on the entire 80% of the outer loop training plus validation data, and the mean AUROC results were reported on the test data of the outer loop. The outer loop was iterated 5 times, each time selecting a different 20% for test data, until all the samples were left out and tested. This whole process was repeated 7 times, each with different random undersampling seeds, where in each of the 7 iterations, 5 AUROC were reported from the outer CV loop, giving a total of 35 values. The mean and SD of the AUROC values were used as the final metrics in this study to measure performance.

### Feature Selection

During the construction of the model, a subset of features was selected from the features listed in Table 1. The goal of feature selection was to avoid using duplicate information (where the same information was present in different features) and maximize the prediction performance of our model.

To avoid the use of duplicate information, we first calculated the intercorrelations between all the features presented in Table 1. We then used only one of each pair of the highly correlated (*r*>0.8) features.

In the model construction, it might not always be true that using all the available features maximizes the prediction accuracy; doing so may actually reduce accuracy owing to overfitting [39]. Thus, to maximize the prediction performance of our model, we selected a subset of features using the following method: we began with the single feature that had the highest correlation with the GAD-7 and then measured the prediction performance of a trained model (on a validation data) using only that feature. Subsequent features were then added one-by-one in order of correlation until all the significant features (presented in Table 1) were used (ie, until adding 1 more feature no longer improved the prediction performance).

### Statistical Analysis

To evaluate the performance of the prediction models, the mean AUROC of the 35 models was compared with the mean AUROC of a model that made a random prediction (ie, mean AUROC close to 0.5) using a modified 1-tailed *t* test developed by Bouckaert and Frank [40]. The modified *t* test considers the fact that the individual AUROC values are not independent from each other, whereas in the original *t* test, the samples are expected to be independent. In our case, because the AUROC generated from a model shared some training data (owing to multiple undersampling and the 5-fold CV) with another, the AUROC values were not independent of each other. In our results, we considered a statistically significant difference at a *P* value significance level of .05.

### Ethics Approval

This study was approved by the University of Toronto Research Ethics Board (protocol #37584).

## Results

### Recruitment and Data Inclusion

A total of 2000 participants provided acceptable submissions from November 23, 2020, to May 28, 2021, and thus received payments. We reviewed the input data and audio for quality and included 1744 participants in the analysis. A detailed description of recruitment and data quality filtering was provided in our previous study [11].

### Data Overview and Demographics of Participants

Of the 1744 participants, 540 (30.96%) were above the GAD-7 screening threshold of 10 and 1204 (69.04%) were below the GAD-7 screening threshold of 10. Hereon, we will refer to those participants with a GAD-7 score ≥10 as the *anxious class* and those with a GAD-7 score <10 as the *nonanxious class*.

Table 2 shows participant demographics obtained from the Prolific recruitment platform. Column 1 of Table 2 provide the names of demographic attributes and each category, while columns 2 and 3 give the number (and percentage) of participants with that attribute in the anxious and nonanxious groups, respectively. The last column gives the *P* values for the chi-square test of the null hypothesis that the difference in categories is independent, to determine if there is a significant difference between the anxious and nonanxious groups, for each categorical factor.

**Table 2:** Demographics split by anxious and nonanxious label and chi-square test (N=1744).

Demographic factors			Anxious, n (%) (n=540)		Nonanxious, n (%) (n=1204)		Chi-square test, *P* value	
**Sex**								<.001
	Male	229 (26)		653 (74)				
	Female	311 (36.1)		551 (63.9)				
**Self-reported ongoing mental health illness or condition**								<.001
	Yes	297 (48.8)		311 (51.2)				
	No	243 (21.4)		893 (78.6)				
**Personal income (£^a^)**								<.001
	<10,000	181 (39.2)		281 (60.8)				
	10,000-19,999	112 (35)		208 (65)				
	20,000-29,999	92 (26.2)		259 (73.8)				
	30,000-39,999	60 (24.6)		184 (75.4)				
	40,000-49,999	36 (24.8)		109 (75.2)				
	50,000-59,999	20 (21.3)		74 (78.7)				
	≥60,000	39 (30.5)		89 (69.5)				
**Age (years)**								<.001
	18-19	27 (38)		44 (62)				
	20-29	239 (38.7)		379 (61.3)				
	30-39	162 (32.7)		334 (67.3)				
	40-49	67 (23.4)		219 (76.6)				
	50-59	39 (22.8)		132 (77.2)				
	≥60	6 (5.9)		96 (94.1)				

^a^1 £=US $1.37.

### Classification Model Performance

In this section, the mean AUROC of a binary classification model that classified between anxious and nonanxious classes is presented. The following subsections summarize our main empirical results for different types of inputs to the classification models.

#### Acoustic and Linguistic Features as Input

The mean AUROC for the model constructed using a subset of the acoustic and linguistic features selected using the feature selection method described in the Methods section is reported. As described in the Methods section, the features that contain very similar information were not used based on the intercorrelation between the features. Table 3 presents the features with high intercorrelation (*r*>0.8) between the features presented in Table 1. We only considered using one of each pair of the highly intercorrelated features.

**Table 3:** Features with high intercorrelation (similar features) with each other.

Sample and feature			*r*		*P* value
**All samples**					
	AllPunc, Period	0.93		<.001	
	i, ppron	0.81		<.001	
**Female samples**					
	AllPunc, Period	0.93		<.001	
	Intensity_mean, intensity_std	0.93		<.001	
**Male samples**					
	AllPunc, Period	0.93		<.001	

**Figure 1:**
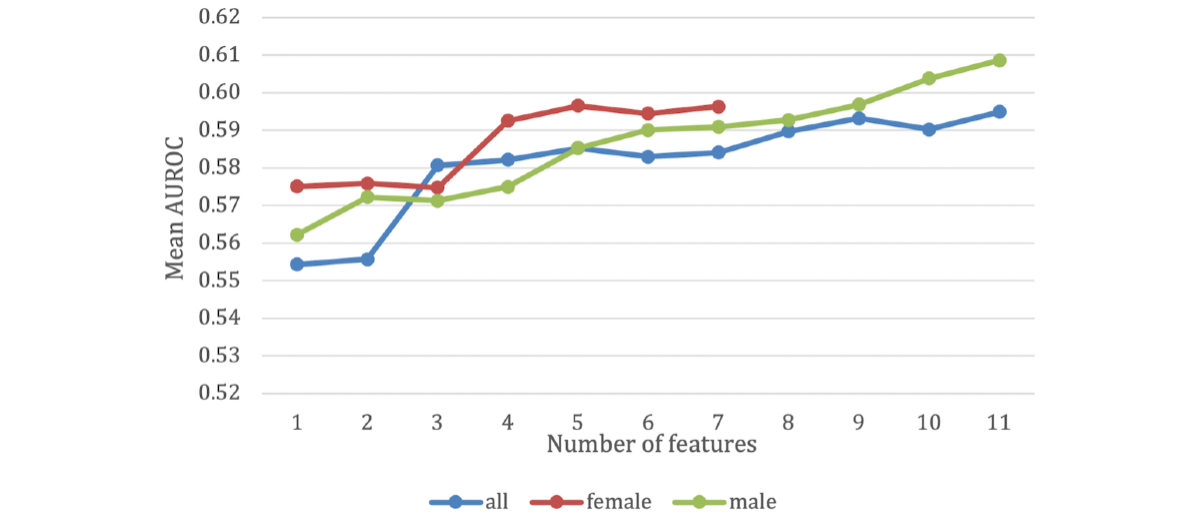
Mean area under the receiver operating characteristic (AUROC) as a function of the number of selected features.

As described in the *Methods* section, the acoustic and linguistic features starting with the feature with the highest correlation were included in the model, incrementally, if they showed improvement in the performance of the model. Table 4 shows the subset of features used for the 3 data sets, and Figure 1 shows the mean AUROC as a function of the number of selected features. Using the feature selection method discussed in the Methods section, the number of features required to produce the maximum mean AUROC was 11 for the all-sample, 7 for the female sample, and 11 for the male sample data set, as shown in Table 4. The best model is the one that included all the features listed in Table 4 (according to the data set).

Table 5 shows the mean AUROC across the 35 data splits, as described in the Methods section. It also provides results of the 1-tailed *t* test comparison of the best model with that of a random model.

**Table 4:** Subset of acoustic and linguist features used in the 3 models after feature selection.

Feature	Data set					
	All-sample		Female sample		Male sample	
	*r*	*P* value	*r*	*P* value	*r*	*P* value
AllPunc	0.13	<.001	—^a^	—	—	—
Assent	0.1	<.001	—	—	0.11	.001
Relativ	−0.09	<.001	—		−0.1	.002
Motion	−0.08	<.001	−0.1	.003	—	—
mfcc_std_2	−0.08	.002	—	—	—	—
mfcc_std_3	−0.07	.002	—	—	—	—
Focusfuture	−0.07	.003	—	—	−0.08	.02
mfcc_std_5	−0.06	.01	—	—	—	—
Death	0.06	.01	0.07	.04	—	—
See	−0.06	.01	−0.09	.006	—	—
mfcc_std_4	−0.05	.045	—	—	—	—
Period	—	—	0.16	<.001	—	—
Dic	—	—	−0.08	.02	—	—
Power	—	—	0.07	.03	—	—
lpcc_std_10	—	—	−0.07	.03	—	—
speaking_duration	—	—	—	—	−0.13	<.001
Leisure	—	—	—	—	0.1	.002
Time	—	—	—	—	−0.1	.004
Ppron	—	—	—	—	0.09	.01
Negemo	—	—	—	—	0.08	.01
Article	—	—	—	—	−0.08	.01
mfcc_mean_5	—	—	—	—	−0.08	.01
f1_mean	—	—	—	—	0.07	.047

^a^Not available because the correlation was not significant.

**Table 5:** Mean area under the receiver operating characteristic (AUROC) of a model trained using the subset of features (N=1744).

Data set	AUROC, mean (SD)	*t* test (*df*)	*P* value
All-sample	0.59 (0.02)	4.93 (34)	<.001
Female sample (n=862)	0.60 (0.04)	4.25 (34)	<.001
Male sample (n=882)	0.61 (0.06)	4.21 (34)	<.001

#### Using Participants’ Demographics

This section presents the performance of the model when augmented with age, sex, and income demographic information. Table 6 shows the mean AUROC of the LR model that used both demographic information and acoustic and linguistic features. It also included a modified *t* test comparison with a random model.

Table 7 shows the results of the *t* test between the model with only acoustic or linguistic features and the model that also used demographic information. Table 8 separates each of the demographic features and shows the mean AUROC of these models when using a single demographic at a time, together with the acoustic and linguistic features.

**Table 6:** Mean area under the receiver operating characteristic (AUROC) of a model trained using demographic information (age, sex, and income) in addition to the acoustic and linguistic features and comparison with a random model (N=1744).

Data set	AUROC, mean (SD)	*t* test (*df*)	*P* value
All-sample	0.64 (0.03)	6.21 (34)	<.001
Female sample (n=862)	0.66 (0.04)	5.89 (34)	<.001
Male sample (n=882)	0.62 (0.07)	4.36 (34)	<.001

**Table 7:** Comparison of model trained using only acoustic or linguistic features with model that also uses demographic information (N=1744).

Data set	AUROC^a^, mean (SD)			*t* test (*df*)		*P* value
	Acoustic and linguistic features	Demographics, acoustic and linguistic features				
All-sample	0.59 (0.02)	0.64 (0.03)	4.01 (34)		<.001	
Female sample (n=862)	0.60 (0.04)	0.66 (0.04)	4.21 (34)		<.001	
Male sample (n=882)	0.61 (0.06)	0.62 (0.07)	0.76 (34)		.45	

^a^AUROC: area under the receiver operating characteristic.

**Table 8:** Mean area under the receiver operating characteristic (AUROC) of the model when adding a single demographic characteristic to the acoustic and linguistic features (N=1744).

Data set	AUROC of model using acoustic and linguistic features and including o*nly one of the demographics,* mean (SD)		
	Age	Income	Sex
All-sample	0.64 (0.03)	0.6 (0.02)	0.59 (0.02)
Female sample (n=862)	0.66 (0.04)	0.6 (0.05)	N/A^a^
Male sample (n=882)	0.62 (0.07)	0.61 (0.06)	N/A

^a^N/A: not applicable.

## Discussion

The main objective of this study was to investigate the prediction performance of a model that screens for GAD from acoustic and linguistic features of impromptu speech. To do so, we have explored an LR model, and in the following subsections, we discuss the findings presented in the *Results* section, as well as the limitations of this study.

### Principal Findings

#### Recruitment and Data Inclusion

As the study continued from November 23, 2020, to May 28, 2021, the recruitment took place during the global COVID-19 pandemic. We speculated that this might have resulted in an above-normal number of participants who work remotely using their personal computers, hence making web-based recruitments relatively quicker.

#### Demographics of Participants

The percentage of anxious and nonanxious participants shows that the anxious group made up to 30.96% (540/1744) of the total, which is much higher than the general population rate of 10% [2]. Previous studies [11,26-28,41] that also used participants recruited from Prolific showed a higher number of anxious participants in the recruitment pool. Table 2 sheds some light on this difference, showing that a similarly high fraction of participants self-reported on their Prolific profile that they have an ongoing mental health condition.

We also aimed to obtain broader demographics than those in the prior study. Most prior studies focused on a certain type of demographics, such as the study by McGinnis et al [13], which focused on children, and the studies by Week et al [17], Salekin et al [20], and Rook et al [23], which focused on undergraduate students. Both these types significantly limited the age range of the participants. The data presented in Table 2 show that the age range of our participants had a broader distribution. The same is true for personal income, which showed a range of economic status in our participant pool.

#### Acoustic and Linguistic Features as Input

The LR used statistically significant acoustic and linguistic features selected by the feature selection method discussed in the *Methods* section and presented in Table 4. Although the correlation between the features used and the GAD-7 was very small (the highest being 0.13), the model built using these features was able to perform significantly better (with *P*<.05) than a random model. The mean AUROC results presented in Table 5 suggest that there is some signal to be detected from the combined effect of the acoustic and linguistic features of speech.

Although it is possible to use the GAD-7 scale to screen for GAD (it has a sensitivity of 89% and a specificity of 82% [12]), it cannot serve the purpose of our study, which is a continuous and passive monitoring of a participant. By contrast, an automated screener that listens passively to speech has the potential to frequently monitor speech samples from participants. Furthermore, the probability of correct prediction can be improved by using multiple measurements under the assumption that each measurement from different speech is relatively independent.

This enhanced accuracy could be achieved by considering the model’s native accuracy as follows: let the accuracy of a correct prediction from a single measurement be *a*, and we take *N* successive measurements, based on *N* successive speech samples, using our model. As a decision procedure, we would decide that most of the measurement is correct—whichever result, anxious or nonanxious, happens in more than *N/2* of the measurements. We were interested in the probability that this decision procedure will produce a correct result. The probability that *n* or more of the *N* measurements would have a correct prediction can be calculated using the cumulative binomial distribution function (Inline graphic 1). Given the decision procedure of taking the correct result to be the majority result of the *N* trials, we set the value of n to be *N/2*, which computes the probability of more than *N/2* correct answers. As long as the single prediction *a* is >0.5, the computed probability *A* will be >*a*. It should be noted that this result does rely on the assumption that the measurements are independent when, in reality, they are not because the measurements were taken from the same person. However, the set of words coming from the person was different, and more spaced-out measurements might have reduced the dependency between the samples. To summarize, it is possible to increase the accuracy of correct predictions by taking multiple measurements and taking the class (anxious or nonanxious) that has been predicted most of the time as the final predicted value.

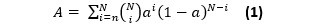



#### Participants’ Demographics as Input Features

In a scenario in which an anxiety screening or prediction model can be deployed, an individual’s demographic information can easily be collected. Thus, it is reasonable to explore the predictive capability of this additional information. Table 6 shows that a model built using demographic information as input in addition to the acoustic and linguistic features was still able to perform significantly better than a random model. Table 7 compares this model with a model that used only acoustic and linguistic features as input. The results show that the demographic information significantly improved the mean AUROC of the models built on the all-sample and female sample data sets but failed on the model built on the male sample data set.

The impact of each demographic variable was explored separately. Table 8 shows the mean AUROC of the model when only one of the demographics was used together with the acoustic and linguistic features. The addition of age affected the prediction performance of the model, whereas the addition of either sex or income did not show a significant improvement. In addition, the fact that the addition of age affected the prediction performance for the model built on female participants suggests that their anxiety depends on age compared with anxiety in male participants.

Comparing our results with a prior study that aimed to predict GAD, there were studies that achieved the above-random prediction for GAD [21,23], and there were studies that did not [28]. Our models performed significantly better than the random model, and we speculated that this might be attributed to the larger and demographically broad sample size that enabled our model to learn a large amount of information in predicting GAD. We also note that a prior study that did not succeed in predicting GAD [28] did, in fact, succeed in predicting SAD. They believed that the symptoms of SAD might be more manifested in the participants’ behavior and, therefore speech, compared with GAD. Other studies focusing on SAD have also been successful in above-random prediction [17,20].

### Limitations

A limitation of this study arises from the data collection method used with respect to the scenarios of use that were described in the Introduction section. We suggested that the prediction of anxiety from speech could be applied to passively collected speech data gathered while the patient is going through their daily activities. This could help in automated anxiety screening, treatment monitoring, and relapse detection. However, the data used in this study were actively collected when the participants spoke in front of a camera, and it may be substantially different from such passively collected speech. Future studies could investigate the models that we suggest using passively collected speech.

Another limitation was the use of a web-based participant recruitment method. Individuals willing to work on a web-based participant recruitment platform may be limited to a particular type of demographics in a certain society. For example, we noted that in our recruitment pool, there was a higher percentage of anxious participants compared with the general population. In our study, we sought generalizability, and even though our participants were more diverse in terms of demographics compared with prior studies, it could be more generalizable if we recruited participants from sources other than the web-based recruitment.

Another limitation was the artificial setup used to replicate the original TSST. In the original TSST, participants described their dream job in front of a live panel of judges. Owing to the restrictions that the COVID-19 pandemic had caused, we were not able to recruit participants for an in-person study; instead, we had participants describe their dream job in front of a camera at their own location (with different recording devices). Despite its limitations, this approach also had important benefits because it enabled us to recruit a large number of participants, which would otherwise have been extremely difficult for an in-person study.

### Conclusions

In this study, we developed a model to predict the presence or absence of GAD based on the speech features. These speech features were chosen because prior studies have suggested that they are associated with other types of anxiety disorders including GAD. Our results have shown that it is possible to achieve the above-random prediction accuracy for GAD from the acoustic and linguistic features of speech while using a larger and more generalizable sample size. Prediction accuracy can also be further improved by adding basic demographic information. Even though we have investigated adding 3 different types of demographic variables (age, sex, and income), the most influential variable that showed improvement in prediction accuracy was age.

Furthermore, we have discussed that the results from multiple measurements have the possibility to improve prediction accuracy. Therefore, we recommend that future studies explore the collection of multiple speech samples sampled throughout the day or week and investigate the extent to which the prediction accuracy can be improved. This will allow for the acoustic and linguistic features of speech, together with basic demographic information, to be used in a system to trigger early intervention, monitor treatment responses, or detect relapses.

## Appendix

Multimedia Appendix 1 Description of the acoustic and linguistic features.

## Abbreviations

AUROC: area under the receiver operating characteristic
BAI: Beck Anxiety Inventory
BIS/BAS: behavioral inhibition/behavioral approach scale
CV: cross-validation
F0: fundamental frequency
GAD: generalized anxiety disorder
GAD-7: Generalized Anxiety Disorder 7-item
LIWC: Linguistic Inquiry and Word Count
LR: logistic regression
SAD: social anxiety disorder
SVM: support vector machine
TSST: Trier Social Stress Test
