# Supplementary material for: Screening for Generalized Anxiety Disorder From Acoustic and Linguistic Features of Impromptu Speech: Prediction Model Evaluation Study
Source: JMIR Form Res. 2022 Oct 28;6(10):e39998. doi: 10.2196/39998 (PMC9652731; doi:10.2196/39998)
Supplement: Multimedia Appendix 1 [file formative_v6i10e39998_app1.pdf]

Description of the acoustic and linguistic features

Mel Frequency Cepstral Coefficients (MFCC): Coefficients derived from a mel-scale cepstral representation of an audio signal. We include 13 MFCCs, a common set of acoustic signals that are designed to reflect changes in perceivable pitch. Descriptive statistics (mean and standard deviation) of the 13 MFCC features were used in the current study. Note that not all MFCC features included in the current study were determined to be significant in prior work; however, these 13 are most commonly assessed together so we included them all as features of interest. The parameters we used when extracting these 13 MFCC features are: window length = 2048 samples; length of FFT window = 2048 samples; samples advance between successive frames = 512 samples; Window type = Hanning; Number of Mel bands = 128.

Linear Prediction Cepstral Coefficients (LPCC): Coefficients derived from a linear prediction cepstral representation of an audio signal. The first 13 cepstrum coefficients are used here. Descriptive statistics (mean and standard deviation) of the 13 LPCC were used in the current study.

Speaking duration: The amount of time, in seconds, that speech was present

Word Count (WC): The total number of words present in the speech-to-text transcript,

Fundamental frequency (F0): is the frequency at which the glottis vibrates or, also known as *pitch* of the voice. The fundamental frequency varies throughout a person's speech, so both the mean and standard deviation of F0 are used as features.

F1: The first formant. It is a frequency peak in a spectrum which have high degree of energy. Typically, the first formant is around 500Hz. The mean and standard deviation were used as features.

Shimmer: The cycle-to-cycle amplitude variation of the sound wave.

Intensity: The mean squared of the amplitude of the sound wave within a given frame. Since the amplitude of a sound wave varies during speech, the mean and standard deviation were used as features.

Linguistic features: Linguistic Inquiry and Word Count (LIWC) was used to acquire the linguistic features. LIWC is based on the counts of words in different pre-set categories. The recent LIWC version (LIWC2015) is made up of a Dictionary of almost 6,400 words. Each of these words belongs to one or more word categories. Given a certain transcript from a participant, LIWC will categorize each of the words in that transcript in one or more pre-set categories based on which category that word belongs to. To apply the LIWC dictionaries, one simply counts the number of words that belong to each category, and each count becomes a feature. There are a total of 93 categories in the LIWC, but not all are relevant for a speech-to-text transcript. We have removed those features that are not relevant - for example informal language words such as 'lol' and 'btw.' Other excluded categories include those relating to some punctuation (e.g., colons, quotation marks, parentheses). Removing these, a total of 80 linguistic features remained.
